# Use of artificial intelligence in Ecuadorian companies

**DOI:** 10.3389/frai.2026.1814070

**Published:** 2026-05-28

**Authors:** Maximiliano Perez-Cepeda, Mauricio Carvache-Franco, Miguel A. Bustamante-Ubilla, Orly Carvache-Franco, Wilmer Carvache-Franco

**Affiliations:** 1 Universidad Católica de Santiago de Guayaquil, Guayaquil, Ecuador; 2 Universidad Bolivariana del Ecuador, Durán, Ecuador; 3 Graduate School of Business, Universidad ESAN, Lima, Peru; 4 Facultad de Ciencias Empresariales, Universidad de Talca, Dos Norte, Talca, Chile; 5 Sistema de Posgrado, Universidad Católica de Santiago de Guayaquil, Guayaquil, Ecuador; 6 Universidad Espíritu Santo, Samborondón, Ecuador; 7 Facultad de Ciencias Sociales y Humanísticas, Escuela Superior Politécnica del Litoral, Guayaquil, Ecuador

**Keywords:** artificial intelligence, Ecuador, emerging economies, perceived ease of use, perceived usefulness, technology acceptance model

## Abstract

**Introduction:**

Artificial intelligence (AI) has become increasingly embedded in organizational processes; however, the mechanisms explaining its adoption are not yet fully understood, particularly in emerging economy contexts. This study examines the relationship between perceived usefulness (PU) and perceived ease of use (PEOU) in the adoption of AI among firms located in Guayas, Ecuador.

**Methods:**

Drawing on the Technology Acceptance Model (TAM), the study analyzes both the conventional directional effect of PEOU on PU and the potential existence of a reciprocal association between these constructs, considering the adaptive nature of AI systems. A quantitative research design was applied using survey data collected from 398 managers of medium and large firms. The analysis was conducted through exploratory and confirmatory factor analyses, followed by structural equation modeling.

**Results:**

The findings indicate that perceived ease of use is positively associated with perceived usefulness, consistent with TAM. In addition, perceived usefulness is also positively associated with perceived ease of use, suggesting a reinforcing interaction between the two constructs. These results are consistent with a dynamic interpretation of AI adoption, where user interaction and system responsiveness may evolve together over time.

**Discussion:**

This study contributes to the literature by extending TAM to AI contexts through a reciprocal perspective and by providing empirical evidence from an underexplored emerging economy. From a managerial perspective, the findings highlight the importance of combining usability and value delivery in AI system design. Given the cross-sectional nature of the data, the results should be interpreted with caution, as causal relationships cannot be established.

## 1 Introduction

AI is increasingly recognized as a key technological driver shaping organizational processes and decision-making across industries. Its capacity to analyze large-scale data, generate predictive insights, and support automated decision processes has positioned AI as a critical enabler of operational efficiency and competitive advantage.

Despite these advantages, the adoption of AI in companies remains uneven and not fully understood. While AI technologies offer significant potential, organizations often face challenges related to implementation complexity, skill gaps, and uncertainty about the actual benefits derived from their use. As a result, understanding the factors that influence AI adoption has become a critical research priority, particularly in emerging economies where technological capabilities and organizational readiness may vary significantly.

The Technology Acceptance Model (TAM) has been widely used to explain the adoption of digital technologies, including AI. Within this framework, two core constructs—perceived usefulness and perceived ease of use—are consistently identified as key determinants of technology acceptance. Perceived usefulness refers to the extent to which individuals believe that using a technology enhances their performance, while perceived ease of use reflects the degree to which the technology is perceived as effortless to use. Traditionally, TAM posits a unidirectional relationship in which greater usability tends to enhance the perceived value of a technology by reducing cognitive effort and improving task efficiency.

Nevertheless, the growing complexity and adaptive capabilities of AI systems suggest that traditional assumptions of technology adoption models may not fully capture the dynamics of AI use. Unlike conventional technologies, AI systems often involve continuous interaction, iterative feedback, and adaptive responses between users and algorithms. In such environments, perceptions of usefulness may evolve through experience, reinforcing user engagement and potentially influencing perceptions of ease of use over time. This opens the possibility of a reciprocal relationship between these constructs, which remains underexplored in empirical research.

From a theoretical perspective, this reciprocal dynamic can be understood through the lens of organizational learning and learning-by-doing mechanisms. As users interact with AI systems, they develop knowledge, skills, and confidence, which reduce perceived complexity and increase ease of use. Simultaneously, as users recognize the tangible benefits of AI in improving performance and decision-making, their perception of usefulness is reinforced, creating a feedback loop between the two constructs. Despite its theoretical plausibility, empirical research examining this bidirectional relationship in the context of AI adoption remains limited.

This gap is particularly relevant in emerging economies such as Ecuador, where firms are increasingly adopting AI technologies but face unique structural, technological, and organizational constraints. In such contexts, understanding the interaction between perceived usefulness and ease of use is essential for explaining adoption behavior and designing effective AI implementation strategies.

Therefore, the objective of this study is to examine the relationship between perceived usefulness and perceived ease of use of AI in entrepreneurial companies in Guayas, Ecuador, with a particular focus on their potential reciprocal influence. Using a quantitative approach based on structural equation modeling, this study seeks to provide empirical evidence on the dynamics between these constructs and contribute to the extension of TAM in the context of AI adoption.

This research contributes to the literature in three main ways. First, it extends the traditional TAM framework by exploring the potential bidirectional relationship between perceived usefulness and ease of use in AI contexts. Second, it provides empirical evidence from an emerging economy, addressing the lack of studies in such settings. Third, it offers practical insights for managers and policymakers seeking to promote effective AI adoption by emphasizing the role of learning processes and user interaction in shaping technology perceptions.

## 2 Theoretical framework and hypotheses development

### 2.1 Technology acceptance model in the context of artificial intelligence

The Technology Acceptance Model (TAM) is one of the most widely recognized frameworks for explaining how individuals adopt and use new technologies (Davis, 1989). The model proposes that technology acceptance is primarily driven by two key constructs: perceived usefulness (PU) and perceived ease of use (PEOU). Perceived usefulness refers to the extent to which individuals believe that a technology can enhance their job performance, while perceived ease of use reflects the degree to which interacting with the system is perceived as effortless.

Over time, TAM has been extensively validated across a wide range of technological settings, including e-commerce, information systems, and digital platforms (Venkatesh and Davis, 2000; Chen and Aklikokou, 2020). More recently, it has also been applied to the study of artificial intelligence (AI) adoption within organizational contexts (Kelly et al., 2023; Na et al., 2022). Empirical findings consistently indicate that perceived usefulness remains a central determinant of AI adoption, whereas perceived ease of use facilitates user interaction with AI-driven systems (Alzoubi, 2024; Wang et al., 2023).

Despite its widespread applicability, TAM was originally conceptualized in relation to relatively stable and deterministic technologies. In contrast, AI systems are inherently adaptive, data-driven, and capable of evolving through user interaction, thereby introducing additional complexity into the adoption process (Enholm et al., 2022; Loureiro et al., 2021). These characteristics suggest that traditional TAM assumptions may need to be reconsidered or extended to adequately capture the dynamic nature of AI adoption.

### 2.2 Perceived ease of use and perceived usefulness in AI adoption

Within the TAM framework, perceived ease of use has consistently been identified as a key antecedent of perceived usefulness. Technologies that are easier to operate tend to reduce cognitive effort and improve user efficiency, thereby increasing their perceived value (Venkatesh and Davis, 2000; Caffaro et al., 2020). This relationship has been empirically supported across various domains, including digital services, e-learning environments, and business applications (Chen and Aklikokou, 2020; Gupta et al., 2021).

In the context of AI, ease of use becomes particularly significant due to the inherent complexity of these systems. AI applications often involve advanced functionalities such as predictive analytics, recommendation engines, and automated decision-support tools. When these technologies are designed with intuitive interfaces and accessible features, users are more likely to perceive them as beneficial and relevant to their tasks (Wang et al., 2023; Alzoubi, 2024).

At the same time, perceived usefulness plays a fundamental role in shaping user attitudes toward AI adoption. AI solutions that clearly demonstrate improvements in decision-making, operational efficiency, and overall performance are more likely to be adopted and sustained within organizational environments (Davenport, 2018; Ocran et al., 2024). In this regard, perceived usefulness not only influences initial adoption decisions but also supports continued usage and engagement with AI technologies.

### 2.3 A reciprocal perspective: learning and feedback effects in AI systems

While the influence of perceived ease of use on perceived usefulness has been extensively documented in prior research, the inverse relationship has received comparatively limited attention, particularly within the context of artificial intelligence. The specific characteristics of AI technologies suggest that this relationship may not be adequately captured by a strictly unidirectional framework, but rather may involve more complex and interactive dynamics.

Beyond individual learning processes, AI systems introduce an additional mechanism through algorithmic adaptation and personalization. By continuously processing user behavior and data inputs, these systems can modify interfaces, recommendations, and decision-support functions in ways that may effectively reduce perceived and objective complexity. This system-driven adaptability distinguishes AI from more static technologies and provides a structural explanation for how perceived usefulness may contribute to perceived ease of use, beyond user familiarity alone.

AI technologies are inherently interactive and adaptive, frequently evolving through ongoing data input and user engagement (Enholm et al., 2022). Such interaction can create conditions that facilitate user learning, allowing individuals to progressively develop familiarity, competence, and confidence in using these systems. From an organizational learning perspective, repeated engagement with technological tools may enhance user capability and lower perceived difficulty, thereby reinforcing perceptions of ease of use (Howard, 2019; Loureiro et al., 2021).

At the same time, as users recognize the practical benefits of AI—such as improved efficiency, greater accuracy, and enhanced decision-making support—their perception of usefulness tends to increase. This perceived value may encourage deeper interaction with the system, which in turn can support further learning and reduce perceived complexity. Such a pattern is consistent with learning-by-doing perspectives, where experience and perceived benefits interact over time (Davenport, 2018).

Recent research on AI adoption indicates that user interaction and perceived value are closely interconnected, pointing to the possibility of feedback effects between perceived usefulness and perceived ease of use (Kelly et al., 2023; Schwaeke et al., 2024). Nevertheless, empirical studies explicitly examining these reciprocal dynamics remain limited, particularly in the context of emerging economies.

### 2.4 Hypotheses development

Based on the theoretical arguments presented above, this study proposes the following hypotheses:

*H1*: Perceived ease of use of artificial intelligence positively influences perceived usefulness of artificial intelligence.

This hypothesis is consistent with the traditional TAM framework, where ease of use enhances the perceived benefits of a technology (Davis, 1989; Venkatesh and Davis, 2000).

*H2*: Perceived usefulness of artificial intelligence positively influences perceived ease of use of artificial intelligence.

This hypothesis extends TAM by proposing that, in AI contexts, the recognition of benefits derived from AI systems reinforces user engagement and learning, thereby increasing perceived ease of use (Davenport, 2018; Enholm et al., 2022).

### 2.5 Research model

Based on the proposed hypotheses, the research model incorporates both traditional and reciprocal relationships between perceived usefulness and perceived ease of use. This model extends TAM by introducing a bidirectional perspective that reflects the dynamic interaction between users and AI systems.

The model is tested using structural equation modeling to evaluate the strength and significance of the relationships between constructs.

## 3 Methodology

### 3.1 Population and sample

The study population consisted of all registered companies, estimated at between 70,000 and 80,000, headquartered in the province of Guayas and registered with the Superintendency of Companies, Securities and Insurance (SUPER CIAS). This entity is a technical body with administrative and economic autonomy, responsible for supervising and controlling the organization, activities, operation, dissolution, and liquidation of companies and other entities, in accordance with the circumstances and conditions established by law. Therefore, it can be inferred that a large number of companies conducting commercial transactions in Ecuador are registered with SUPER CIAS.

As an initial step, some preliminary analysis procedures were applied to calculate the *a priori* sample size using the G*Power 3.1 program, considering a finite population to define the power and effect size parameters for the sample group (Lorenzo-Seva and Ferrando, 2013). Consequently, considering a skewness of 0.017, a probabilistic error of 0.05, and a reliability or probabilistic impulse of 0.95, an a priori sample of 366 cases was calculated. Furthermore, the minimum number of contacts per item or question in the study (n/*p* = 10:1; n/*p* = 5:1) was taken into account to determine the minimum sample size (Freiberg et al., 2013).

Consequently, based on the a priori sample, the n/p criterion, and in accordance with the authorizations for accessing official information from the company registries at SUPER CIAS, a non-probabilistic convenience sampling method was employed, resulting in a final sample of 398 valid responses, exceeding the a priori sample obtained from managers. These responses represent the effective dataset used for the empirical analysis.

The study focused on middle and upper management of medium and large companies located in Guayas, Ecuador.

### 3.2 Procedure

Contacts were randomly selected as typical respondents from the business contexts of Guayas, Ecuador, forming a relevant sample (Izquierdo et al., 2014). Access to the sample was achieved through various virtual channels. During the pilot phase of the instrument, the questionnaire was administered to a small group of 35 executives, ensuring that these individuals were not included in the final study sample (Beavers et al., 2013). The final fieldwork was conducted by the research team using virtual methods, which allowed for contacting an adequate number of respondents, with an average response time of 25 to 35 min.

Data collection was conducted between April 2022 and May 2022 in accordance with the legal requirements related to anonymity and confidentiality. The information was obtained through structured surveys administered to managers of medium and large companies in the province of Guayas.

### 3.3 Research instrument

The instrument used is based on previous studies examining the TAM model (Alraja and Aref, 2015; Barnes et al., 2007; Glover and Benbasat, 2010), shaping a questionnaire that includes 13 variables distributed across two factors, which structure the construct of technology acceptance for artificial intelligence. This analysis aims to update the relevant variables and factors (ease of use and perceived usefulness), taking into account societal evolution and the recent changes companies are facing.

Consequently, the development of this study was structured in two parts. The first part involved administering a questionnaire containing items that respondents needed to answer. To ensure unidirectionality of the items, statements were phrased positively, with responses collected using a 5-point Likert scale. The second part was dedicated to collecting demographic data of the sample for proper characterization.

The present study involved human participants (company managers) and was approved by the Ethics Committee of the Catholic University of Santiago de Guayaquil under Code SINDE-01486-2017. All procedures were conducted in accordance with institutional and national regulations, ensuring anonymity, confidentiality, and informed consent.

### 3.4 Exploratory factor analysis

An Exploratory Factor Analysis (EFA) was initially conducted to examine the underlying structure of the items included in the artificial intelligence acceptance questionnaire. Subsequently, a Confirmatory Factor Analysis (CFA) was performed to assess the relationship between observed variables and their corresponding latent constructs (Garson, 2013).

Factor loadings were used as indicators of the strength of association between items and their respective dimensions. Higher loading values indicate stronger relationships, and, in line with commonly accepted empirical criteria, loadings of 0.70 or above were considered desirable, although lower thresholds may be acceptable depending on theoretical justification and model performance (Raubenheimer, 2004). This approach ensures that the retained indicators contribute meaningfully to the construct representation (Blume, 1976; Barclay et al., 1995).

The factor extraction process was carried out using principal component procedures (Timmerman and Lorenzo-Seva, 2011), based on the Pearson correlation matrix, followed by varimax rotation to facilitate interpretability of the factor structure (Freiberg et al., 2013; Lorenzo-Seva and Ferrando, 2013). Given that responses were measured on a five-point Likert scale, the data were assumed to approximate normal distribution properties for analytical purposes (Izquierdo et al., 2014).

For the psychometric evaluation, communalities and explained variance were examined, considering values equal to or greater than 0.50 as acceptable. Internal consistency was assessed using Cronbach’s alpha, with values above 0.80 indicating satisfactory reliability (Zavaleta-de Arma et al., 2020). Additionally, factor loading thresholds of at least 0.60 were applied to ensure the adequacy and representativeness of the identified dimensions.

### 3.5 Confirmatory structural modeling

To further validate the measurement structure, a confirmatory structural modeling approach was implemented (Izquierdo et al., 2014). The original items associated with the artificial intelligence acceptance construct were analyzed to assess the consistency and stability of the latent variables. Preliminary confirmatory procedures were conducted to verify the adequacy of the measurement model (Forero and Gómez, 2017).

Standardized factor estimates and statistical significance levels were evaluated to determine the robustness of the relationships between observed variables and latent constructs (Izquierdo et al., 2014; Marsh et al., 2014). These results were complemented by the use of Exploratory Structural Equation Modeling (ESEM), allowing for a more flexible assessment of the factor structure before confirming the final model through goodness-of-fit indicators (Marsh et al., 2014; Zavaleta-de Arma et al., 2020).

Model fit was assessed using a combination of absolute, incremental, and error-based indices. The chi-square statistic was used to evaluate the overall fit between the observed and estimated covariance matrices (Kline, 2005). Acceptable model fit is generally indicated by chi-square to degrees of freedom ratios between 2 and 3, although values up to 5 may be considered adequate in exploratory contexts (Hair et al., 1999). The Non-Centrality Parameter (NCP) was also considered as an alternative indicator, with values below 2 suggesting acceptable fit (Martínez, 2003).

Additional fit indices included the Goodness-of-Fit Index (GFI), where values equal to or greater than 0.90 indicate satisfactory model adjustment (Torres, 2011). Error-based measures such as the Root Mean Square Residual (RMR) were used to assess discrepancies between observed and estimated values, with lower values indicating better fit (Arbuckle, 2003; Byrne, 2001).

The Root Mean Square Error of Approximation (RMSEA) was also examined, as it reflects how well the model is expected to fit the population covariance matrix (Lévy, 2003). RMSEA values below 0.05 indicate good fit, while values between 0.05 and 0.08 are considered acceptable (Kline, 2005).

Finally, the Comparative Fit Index (CFI) was used as an incremental fit measure, with values of 0.90 or higher indicating acceptable model performance (Muthen and Asparouhov, 2013). The chi-square likelihood ratio (CMIN/DF ≤ 3) was also evaluated alongside RMSEA thresholds to ensure consistency in model fit assessment (Freiberg et al., 2013; Forero and Gómez, 2017).

### 3.6 Software usage

To determine the *a priori* sample size, the G*Power 3.1 program was used. For data analysis, processing, and factor modeling, the statistical software SPSS V23 was employed. Structural covariance modeling, to assess the relationships among factors and their mutual influence, was performed using AMOS from SPSS (Freiberg et al., 2013; Izquierdo et al., 2014). Lastly, as a general rule to minimize the effects of variance on the obtained average data, standardized estimates (0–1) were interpreted, ensuring they fell within the corresponding range of acceptability (Beavers et al., 2013; Lorenzo-Seva and Ferrando, 2013).

## 4 Results

The following section presents the research results, starting with a description of the sample, followed by the factor analysis of the items related to Artificial Intelligence, and concluding with the structural covariance and variance modeling determined in this study.

### 4.1 Sample description

The total number of contacts in the sample was 472, from which a final, cleaned sample of 398 valid cases was obtained (Makowski et al., 2019; Tang et al., 2018). This sample size exceeded the a priori estimate determined using G*Power (Tripathi and Siddiqui, 2018), reducing the error rate (1–0.9639) to 3.61%, below the initially estimated 5%. Table 1 shows a breakdown of the sample companies according to the International Standard Industrial Classification Rev. 4.

**Table 1:** Sample Demographics.

CIIU	Industry sector	Quantity	Percent
70	Professional services	89	22.36%
61	Information and communication technology	45	11.31%
41	Construction	41	10.30%
10	Manufacturing	34	8.54%
47	Retail trade	34	8.54%
81	General services	26	6.53%
64	Financial services	14	3.52%
85	Education	14	3.52%
01,02,03	Agriculture, forestry and fishing	12	3.02%
49–53	Transportation and storage	10	2.51%
46–47	Wholesale and retail trade	9	2.26%
86	Human health activities	8	2.01%
55	Accommodation and food service activities	5	1.26%
80	Private security services	5	1.26%
03.1	Fishing	4	1.01%
73.1	Advertising	4	1.01%
79	Tourism	3	0.75%
06, 07	Energy, oil and gas	3	0.75%
1	Agriculture	2	0.50%
35	Electricity, gas, steam and air conditioning supply	2	0.50%
84	Public administration	2	0.50%
70.2	Business acceleration consulting	2	0.50%
73.11	Social media	2	0.50%
18	Printing services	1	0.25%
21	Pharmaceutical industry	1	0.25%
49	Commercial transportation	1	0.25%
56	Hospitality, food and beverage	1	0.25%
63	Information service activities	1	0.25%
65	Insurance services	1	0.25%
68	Real estate activities	1	0.25%
69	Legal activities	1	0.25%
90	Entertainment activities	1	0.25%
01.4	Livestock farming	1	0.25%
03.2	Aquaculture	1	0.25%
19.2	Fuel production	1	0.25%
29.3	Automotive parts manufacturing	1	0.25%
30.1	Shipbuilding	1	0.25%
32.5	Manufacturing of medical supplies	1	0.25%
33.12	Electrical maintenance	1	0.25%
38.2	Waste incineration	1	0.25%
43.22	Heating and air conditioning services	1	0.25%
47.3	Gas stations	1	0.25%
50.1	Water transport	1	0.25%
69.2	Accounting consulting services	1	0.25%
74.1	Clothing design	1	0.25%
78.10	Migration services	1	0.25%
82.99	Customs brokerage	1	0.25%
86.23	Dental services	1	0.25%
86.9	Clinical laboratories and medical equipment sales	1	0.25%
93.1	Sports	1	0.25%
93.19	Sports services	1	0.25%
	Total	398	100.00%

### 4.2 Factor analysis

Applying the factor analysis procedures, a Kaiser-Meyer-Olkin (KMO) value of 0.923 was obtained, which is above the threshold of 0.60. This indicates that the sample is suitable for data analysis using factor analysis methods. Similarly, Bartlett’s test of sphericity was conducted to determine if the correlation matrix is an identity matrix. The significance index obtained was 0.000, confirming that factor analysis is appropriate for this study (Table 2). Finally, the principal component analysis extracted two well-defined factors that explain 75.946% of the total variance. Consequently, this study established a system of two related factors, including a total of 11 variables with high factor loadings (≥ 0.70), as detailed in Table 2.

**Table 2:** Factor analysis of artificial intelligence components related to entrepreneurship.

Rotated component matrix	Component		
	UP	FUP	F3
Using Artificial Intelligence (AI) would increase my productivity.	0.911		
Using Artificial Intelligence (AI) would make it easier for me to do my job.	0.905		
Using Artificial Intelligence (AI) would increase my work effectiveness.	0.898		
I would find Artificial Intelligence (AI) useful in my job.	0.885		
Using Artificial Intelligence (AI) would improve my job performance.	0.884		
Using Artificial Intelligence (AI) would help me do my tasks faster.	0.856		
My interaction with Artificial Intelligence (AI) would be clear and understandable.		0.890	
Learning to use Artificial Intelligence (AI) would be easy for me.		0.870	
I find Artificial Intelligence (AI) flexible to interact with.		0.835	
It would be easy for me to become an expert in using Artificial Intelligence (AI).		0.810	
It would be easy for me to achieve what I want with Artificial Intelligence (AI).		0.749	
I feel that my ability to determine the ease of use of Artificial Intelligence (AI) is limited by my lack of experience.			0.979
Variance explained by each factor.	59.174	16.772	7.670
Total Variance Explained.	83.616		
Extraction method: principal component analysis. Rotation method: Varimax with Kaiser normalization^a^			
a. The rotation converged in 4 iterations.			

KMO test and Bartlett		
Kaiser-Meyer-Olkin Measure of Sampling Adequacy		0.923
Bartlett’s Test of Sphericity	Approx. Chi-Square	4759.676
	df	66
	Sig.	0.000

Total variance explained									
Component	Initial eigenvalues			Extraction sums of squared loadings			Rotation sums of squared loadings		
	Total	% of variance	% cumulative	Total	% of variance	% Cumulative	Total	% of variance	% Cumulative
1	7.101	59.174	59.174	7.101	59.174	59.174	5.123	42.690	42.690
2	2.013	16.772	75.946	2.013	16.772	75.946	3.874	32.286	74.976
3	0.920	7.670	83.616	0.920	7.670	83.616	1.037	8.640	83.616
Extraction method: principal component analysis.									

Convergent and Discriminant Validity. To assess the reliability and validity of the measurement model, composite reliability (CR) and average variance extracted (AVE) were calculated. The results indicate that all constructs achieved CR values above the recommended threshold of 0.70, confirming internal consistency (Hair et al., 1999). Additionally, AVE values exceeded the minimum criterion of 0.50, indicating adequate convergent validity.

Discriminant validity was evaluated using the Fornell–Larcker criterion (Fornell and Larcker, 1981). The square root of the AVE for each construct was greater than the correlations between constructs, confirming that each construct is empirically distinct.

Table 3 shows Convergent Validity and Reliability, and Table 4 shows Discriminant Validity (Fornell–Larcker Criterion). These results support the robustness of the measurement model and ensure that the latent constructs are both reliable and valid for subsequent structural analysis.

**Table 3:** Convergent validity and reliability.

Construct	Items	CR	AVE
Perceived usefulness (PU)	6	0.96	0.80
Perceived ease of use (PEOU)	5	0.93	0.72

CR, Composite reliability; AVE, Average variance extracted. The results indicate that all constructs exceed the recommended thresholds of CR ≥ 0.70 and AVE ≥ 0.50 (Hair et al., 1999), confirming adequate convergent validity and internal consistency.

**Table 4:** Discriminant validity (Fornell–Larcker criterion).

Construct	PU	PEOU
Perceived usefulness (PU)	0.894	
Perceived usefulness (PEOU)	0.563	0.848

Diagonal elements represent the square root of AVE. Off-diagonal elements represent correlations between constructs.

The square root of AVE for each construct is greater than the inter-construct correlation, confirming discriminant validity according to the Fornell–Larcker criterion (Fornell and Larcker, 1981).

### 4.3 Structural confirmation of the factor model through covariances and variances

**Figure 1:**
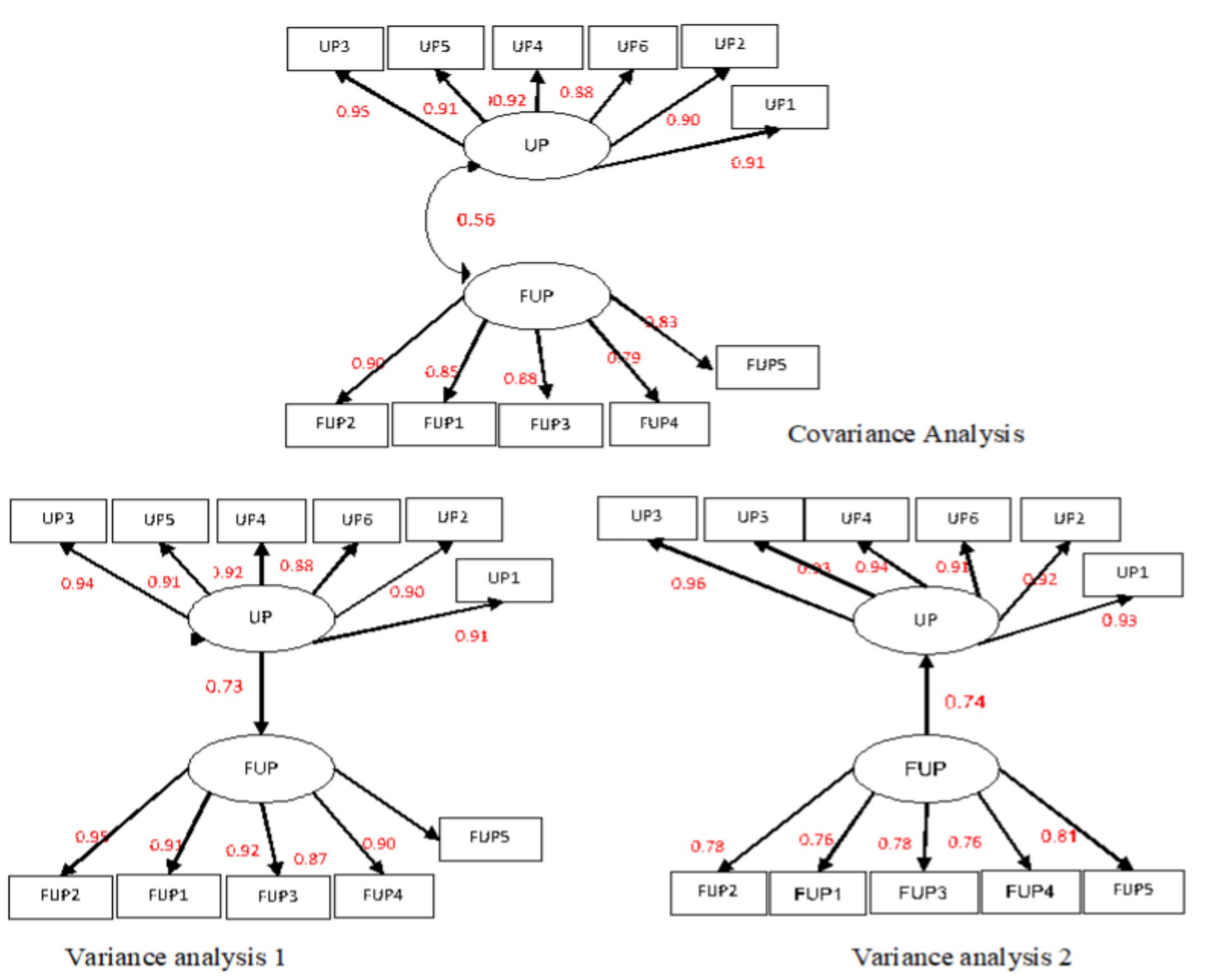
Structural covariance and variance modeling (1 and 2).

Figure 1 presents the proposed analysis of covariance (<-->) that establishes and determines a relationship index between the two factors, shaping an identifiable construct. These factors include a selected group of 11 variables that make up an Artificial Intelligence construct related to the entrepreneurial dimension. It is observed that all factors achieve standardized estimates within the range (0–1), as well as significant unstandardized (p) values (***).

### 4.4 Structural covariance and variance modeling

As shown in Figure 1 and detailed in Table 5, the covariance analysis was conducted to determine the degree of relationship between the two factors under study. The factors Perceived Usefulness (PU) and Perceived Ease of Use (PEOU) are composed of an adequate number of variables (≥ 3) and show significant covariance indices, supporting the internal consistency of the construct.

**Table 5:** Covariance analysis of factors related to artificial intelligence and entrepreneurship.

Standardized regression weights	Estimate	Standardized regression weights	Estimate
UP3 < ---UP	0.945	FUP2 < ---FUP	0.905
UP5 < ---UP	0.914	FUP1 < ---FUP	0.852
UP4 < ---UP	0.923	FUP3 < ---FUP	0.863
UP6 < ---UP	0.882	FUP4 < ---FUP	0.791
UP2 < ---UP	0.900	FUP5 < ---FUP	0.826
UP1 < ---UP	0.911		

Covariances	Estimate	S. E.	C. R.	*p*	Correlations
UP<-- > FUP	0.535	0.057	9.411	***	0.563

Goodness of fit indices for covariance analysis					
Model	NPAR	CMIN	DF	*p*	CMIN/DF
Default model	19	121.284	47	0.000	2.581
Saturated model	66	0.000	0		
Independence model	11	4769.533	55	0.000	86.719

Baseline comparisons	NFI Delta1	RFI rho1	IFI Delta2	TLI rho2	CFI
Default model	0.975	0.970	0.984	0.982	0.984
Saturated model	1.000		1.000		1.000
Independence model	0.000	0.000	0.000	0.000	0.000

Model	RMSEA	LO 90	HI 90	PCLOSE
Default model	0.063	0.049	0.077	0.058
Independence model	0.465	0.454	0.476	0.000

Next, the analysis aimed to determine the mutual influence of these two factors, UP and FUP. First, using variance analysis 1, the impact of the UP factor on the FUP factor was determined, yielding a high and significant index of 0.73***. Similarly, an inverse analysis was conducted to assess the extent to which the FUP factor affects the UP factor. This yielded a relatively higher and also significant estimator of 0.74*** through variance analysis 2.

In addition to the observations made in the previous paragraphs, Table 5, which contains the covariance analysis estimators, details the formation of a coherent system for the factors UP and FUP, including their respective variables and the factorial loadings of each. For instance, the UP factor includes 6 items with loadings ranging from 0.882 (UP6) to 0.945 (UP3), confirming the representativeness of this latent variable. On the other hand, the FUP factor comprises 5 observable variables with factorial loadings ranging from 0.791 (FUP4) to 0.905 (FUP2), validating the relevance of this second factor.

As shown in Table 5, the covariance analysis provides a set of fit indices that confirm the model. Firstly, the CMIN/DF coefficient is 2.581, which falls within the optimal range (≤ 3). Additionally, the Comparative Fit Index (CFI) of 0.984 exceeds the minimum required value (≥ 0.90). Finally, the Root Mean Square Error of Approximation (RMSEA) estimator is 0.063, which is within the acceptable range (0.05 ≤ 0.08) (Freiberg et al., 2013; Forero and Gómez, 2017).

Furthermore, as seen in Table 6, the initial relationship of incidence of the construct was verified through the estimators obtained from the variance analysis. For instance, the factor UP has a significant impact on the factor FUP, with an index of 0.734***.

**Table 6:** Variance analysis of the factors related to artificial intelligence and entrepreneurship.

Standardized regression weights	Estimate	Standardized regression weights	Estimate
UP3 < ---UP	0.943	FUP2 < ---FUP	0.947
UP5 < ---UP	0.911	FUP1 < ---FUP	0.910
UP4 < ---UP	0.919	FUP3 < ---FUP	0.920
UP6 < ---UP	0.880	FUP4 < ---FUP	0.869
UP2 < ---UP	0.897	FUP5 < ---FUP	0.896
UP1 < ---UP	0.910		

Standardized regression weights	Estimate	Factor	Estimate	S. E.	C. R.	*p*
FUP < ---UP	0.734	UP	0.940	0.069	13,664	***

Goodness of fit indices for covariance analysis					
Model	NPAR	CMIN	DF	*p*	CMIN/DF
Default model	29	214.414	48	0.000	4.467
Saturated model	77	0.000	0		
Independence model	22	4769.533	55	0.000	86.719

Baseline comparisons	NFI Delta1	RFI rho1	IFI Delta2	TLI rho2	CFI
Default model	0.955	0.948	0.965	0.960	0.965
Saturated model	1.000		1.000		1.000
Independence model	0.000	0.000	0.000	0.000	0.000

Model	RMSEA	LO 90	HI 90	PCLOSE
Default model	0.093	0.081	0.106	0.000
Independence model	0.465	0.454	0.476	0.000

Additionally, as shown in Table 6, the variance analysis aimed at determining the impact between the factors provides indices of goodness of fit that support the hypothesis proposed in this initial model. The CMIN/DF coefficient obtained is 4.467, which falls within the acceptable range. Although the optimal range for Chi-square/df values is between 2 and 3, a value extending up to 5 is considered adequate (Hair et al., 1999). The Comparative Fit Index (CFI) reached a value of 0.965, exceeding the minimum required threshold of 0.90, indicating a good fit. The Root Mean Square Error of Approximation (RMSEA) has an estimated value of 0.093, which slightly exceeds the acceptable range suggested by the literature (0.05 ≤ 0.08) (Freiberg et al., 2013; Forero and Gómez, 2017).

Finally, as described in Table 7, the variance analysis once again determined the impact of the factor FUP on the factor UP, yielding a significant estimate of 0.74***. This value is slightly higher than the impact found in the reverse analysis previously conducted, which was 0.73***, also significant.

**Table 7:** Analysis of variance of factors related to artificial intelligence and entrepreneurship.

Standardized regression weights	Estimate	Standardized regression weights	Estimate
UP3 < ---UP	0.959	FUP2 < ---FUP	0.776
UP5 < ---UP	0.934	FUP1 < ---FUP	0.760
UP4 < ---UP	0.941	FUP3 < ---FUP	0.776
UP6 < ---UP	0.909	FUP4 < ---FUP	0.759
UP2 < ---UP	0.923	FUP5 < ---FUP	0.811
UP1 < ---UP	0.932		

Standardized regression weights	Estimate	Factor	Estimate	S. E.	C. R.	*p*
UP<---FUP	0.739	FUP	0.715	0.061	11.704	***

Goodness of fit indices for covariance analysis					
Model	NPAR	CMIN	DF	*p*	CMIN/DF
Default model	32	125.447	45	0.000	2.788
Saturated model	77	0.000	0		
Independence model	22	4769.533	55	0.000	86.719

Baseline comparisons	NFI Delta1	RFI rho1	IFI Delta2	TLI rho2	CFI
Default model	0.974	0.968	0.983	0.979	0.983
Saturated model	1.000		1.000		1.000
Independence model	0.000	0.000	0.000	0.000	0.000

Model	RMSEA	LO 90	HI 90	PCLOSE
Default model	0.067	0.053	0.081	0.022
Independence model	0.465	0.454	0.476	0.000

To confirm these findings, Table 7 displays the goodness-of-fit indices that support the proposed second hypothesis of the model. First, the CMIN/DF coefficient is optimal, reaching a value of 2.788, which is clearly within the acceptable range of Chi-square/df between 2 and 3 (Hair et al., 1999). Next, the Comparative Fit Index (CFI) achieves a value of 0.983, which is above the minimum required (≥ 0.90). Finally, the Root Mean Square Error of Approximation (RMSEA) estimator is 0.067, which falls comfortably within the acceptable range (0.05 ≤ 0.08) suggested by Freiberg et al. (2013) and Forero and Gómez (2017).

## 5 Discussion

This study analyzed the association between perceived usefulness (PU) and perceived ease of use (PEOU) in the context of artificial intelligence (AI) adoption among firms in Guayas, Ecuador. The findings provide support for both the traditional directional relationship proposed by the Technology Acceptance Model (TAM) and a potential reciprocal association between these constructs.

First, the findings confirm that perceived ease of use positively influences perceived usefulness. This result is consistent with the original TAM framework (Davis, 1989) and with a large body of empirical studies across different technological contexts (Venkatesh and Davis, 2000; Chen and Aklikokou, 2020). In the context of AI, this suggests that when users perceive AI systems as easy to use—particularly through accessible interfaces and software tools—they are more likely to recognize their value in improving performance and efficiency. This is particularly relevant in business environments where the complexity of AI technologies may otherwise hinder adoption.

Second, the results provide evidence that perceived usefulness also positively influences perceived ease of use. This finding extends the traditional TAM perspective by suggesting that, in AI contexts, the perceived benefits of technology play a role in shaping user experience. When users recognize that AI systems contribute to improved decision-making, productivity, and operational outcomes, they are more motivated to engage with these systems, which in turn facilitates learning and reduces perceived complexity. This result aligns with prior research highlighting the importance of perceived value and user interaction in AI adoption (Davenport, 2018; Enholm et al., 2022).

Rather than testing reciprocity as an independent hypothesis, the reciprocal relationship is interpreted as emerging from the joint significance of H1 and H2, consistent with the analytical approach employed.

Taken together, these findings support the existence of a reciprocal relationship between perceived usefulness and perceived ease of use. This bidirectional pattern suggests that AI adoption may be better understood as a dynamic process, potentially involving iterative interactions between users and technology. The observed bidirectional associations are consistent with theoretical explanations based on learning-by-doing and adaptive interaction mechanisms, rather than constituting direct empirical evidence of such processes (Howard, 2019; Loureiro et al., 2021).

Importantly, this reciprocal relationship should be interpreted with caution. Given the cross-sectional design of the study, the results do not imply strict causality but rather indicate statistically significant associations that suggest mutually reinforcing dynamics. Future research using longitudinal or non-recursive structural equation models would be necessary to fully validate these feedback effects.

From a contextual perspective, the findings contribute to understanding AI adoption in emerging economies. In environments such as Ecuador, where firms may face resource constraints, limited technological infrastructure, and skill gaps, the interaction between perceived usefulness and ease of use becomes particularly relevant. The results suggest that demonstrating the tangible benefits of AI can accelerate user learning and reduce perceived complexity, thereby facilitating adoption even in contexts with structural limitations. This highlights the importance of focusing not only on technological design but also on user experience and value realization.

Finally, compared to prior studies on AI adoption, which have largely focused on linear relationships between TAM constructs, this study provides empirical evidence supporting a more dynamic perspective. By identifying reciprocal effects between perceived usefulness and perceived ease of use, the findings contribute to a deeper understanding of how users interact with AI systems and how these interactions evolve over time.

Overall, the results reinforce the relevance of TAM in explaining AI adoption while also suggesting the need for theoretical extensions that account for the adaptive and interactive nature of AI technologies.

## 6 Conclusion

### 6.1 Theoretical implications

This study contributes to the literature on technology adoption and artificial intelligence in several important ways.

First, it extends the Technology Acceptance Model (TAM) by providing empirical evidence that the relationship between perceived usefulness (PU) and perceived ease of use (PEOU) may not be strictly unidirectional in AI contexts. While prior research has consistently demonstrated that greater usability tends to enhance the perceived value of a technology by reducing cognitive effort and improving task efficiency (Davis, 1989; Venkatesh and Davis, 2000), the findings of this study suggest that perceived usefulness may also influence ease of use. This highlights the need to reconsider traditional assumptions of TAM when applied to adaptive and interactive technologies such as artificial intelligence.

Second, this study introduces a dynamic perspective on technology adoption by incorporating feedback effects between key TAM constructs. The results are consistent with the idea that AI adoption may involve iterative learning processes, as suggested by the observed associations and supported by theoretical perspectives on organizational learning. This interpretation aligns with theoretical perspectives on organizational learning and learning-by-doing, which suggest that repeated interaction with technological systems may reinforce both perceived usefulness and ease of use over time.

Third, the study contributes to the limited body of literature on AI adoption in emerging economies. Most prior research on TAM and AI adoption has been conducted in developed countries. By focusing on entrepreneurial companies in Ecuador, this study provides context-specific evidence that enriches the global understanding of AI adoption and highlights the importance of considering institutional and organizational constraints in emerging markets.

Overall, these contributions suggest that future research on technology acceptance should move beyond static models and incorporate dynamic, interaction-based perspectives, particularly in the context of AI and other advanced digital technologies.

### 6.2 Practical implications

The findings of this study offer several practical insights for managers, organizations, and policymakers seeking to promote the adoption of artificial intelligence.

First, the results indicate that improving the ease of use of AI systems remains a critical factor in enhancing their perceived usefulness. Organizations should prioritize the design and implementation of user-friendly AI interfaces, training programs, and support systems that reduce the complexity of AI technologies and facilitate user interaction.

For example, organizations should prioritize hands-on training workshops that allow users to directly experience the benefits of AI, rather than relying solely on documentation or passive instruction. Such experiential learning approaches can accelerate both perceived usefulness and ease of use.

Additionally, structured onboarding protocols should be implemented, where early-stage interactions emphasize quick wins and visible performance improvements, reinforcing perceived usefulness and facilitating subsequent ease of use.

Given the sectoral diversity observed in the sample, organizations should also tailor AI implementation strategies to specific industry contexts, particularly in sectors with lower digital maturity.

From a policy perspective, institutions in Ecuador could support AI adoption through targeted training programs, sector-specific digital transformation initiatives, and incentives for technological capability development.

Second, the study highlights the importance of demonstrating the tangible benefits of AI adoption. Managers should focus on clearly communicating and evidencing how AI contributes to improved performance, efficiency, and decision-making. When users perceive concrete benefits, they are more likely to engage with the technology, which in turn enhances their perception of ease of use.

Third, the reciprocal relationship identified in this study suggests that AI adoption may be better approached as a learning-oriented process rather than a one-time implementation rather than a one-time implementation. Organizations should create environments that encourage experimentation, continuous use, and knowledge sharing, allowing users to develop familiarity and confidence in interacting with AI systems.

Finally, for policymakers and institutions in emerging economies, the findings underscore the importance of supporting AI adoption through training initiatives, digital infrastructure development, and policies that promote technological literacy. Facilitating both the perceived usefulness and ease of use of AI can accelerate its diffusion across firms and sectors.

### 6.3 Limitations and future research

Certain limitations should be acknowledged when interpreting these findings.

First, the use of a cross-sectional research design limits the ability to draw causal inferences between perceived usefulness and perceived ease of use. Although the findings suggest a reciprocal relationship, longitudinal studies would be required to fully capture the dynamic interactions between these constructs over time.

Second, the analysis was conducted using separate structural models rather than a fully specified non-recursive structural equation model. While this approach provides initial empirical evidence of bidirectional relationships, future research should employ more advanced modeling techniques to formally test reciprocal effects.

Third, the data were collected in 2022. Given the rapid evolution of artificial intelligence technologies, user perceptions and adoption patterns may have changed. Future studies should examine whether the relationships identified in this research remain stable over time or evolve as AI technologies mature.

Fourth, the study focuses on entrepreneurial companies in a specific geographic context (Guayas, Ecuador), which may limit the generalizability of the findings. Future research could replicate this study in different countries, industries, and organizational contexts to validate and extend the results.

Finally, future studies could incorporate additional variables, such as trust in AI, organizational readiness, and technological competence, to develop more comprehensive models of AI adoption.

Despite these limitations, this study provides valuable insights into the dynamics of AI adoption and offers a foundation for future research on technology acceptance in increasingly complex and adaptive technological environments.

## Data Availability

The raw data supporting the conclusions of this article will be made available by the authors, without undue reservation.
